# Prosthetic Dentistry

**Published:** 1894-03

**Authors:** J. C. Brewer


THE
### AMERICAN JOURNAL
—OF—
### DENTAL SCIENCE.

VOL. XXVII. BALTIMORE, MARCH, 1894. NO. 11.

ARTICLE I.

### PROSTHETIC DENTISTRY.

**Read in the Georgia Dental Association.**

REPORTED BY MRS. J. M. WALKER.

Dr. J. C. Brewer, of Blackshear, Georgia, presented an interesting paper on Operative Dentistry. He said:

We all know the importance of properly caring for children's teeth, and the necessity for enlightening parents pertaining to the welfare of the teeth, especially in the distribution of properly prepared literature, giving the public a chance to learn the importance of having a healthy mouth, through the more general distribution of suitable dental literature. It is of the greatest importance to gain the confidence of young patients, and of never misleading them by false promises of painless operations. Cavities in deciduous teeth should be filled with gutta-percha. In the grinding surfaces of molars and bicuspids, the gutta-percha fillings may be capped with an amalgam having a large percentage of tin. For devitalizing the nerve of a deciduous tooth, when this cannot be avoided, we use equal parts of oil of cloves and carbolic acid. We are reminded of the death of a child of seven years of age occasioned by the use of "toothache drops" purchased at a country store. This penetrated through the foramen, necrosis ensued, and in spite of all remedial aid death was the result. The bottom of the small bottle was covered for an eighth of an inch with arsenic. The importance of proper use of the molar teeth in mastication, and the use of food requiring thorough mastication should be urged on all. For permanent fillings we have had very satisfactory results from the use of Daniel's mastic as a lining for large cavities, which forms an impervious coating, closing the mouth of the dental tubuli, and acting as a non-conductor to some extent. For root fillings we use cotton saturated with creasote and dipped in oxid of zinc. We do not consider pulp capping good practice, nor "a safe road to travel," though we have been quite successful in a small per cent. of cases. When there is a thin layer of dentine over the pulp we would fill half or two-thirds full with oxyphosphate. In all operations fair dealing should form the basis of action, keeping in view the elevation of the profession through the relief of suffering humanity.

Dr. W. G. Browne described his method of making plates of pure soft gold directly on the plaster model without the use of metal dies, as given in the report of the Alabama Society.

A patient wearing one of the plates so constructed, was present and ready to have it examined by any one who desired to see for themselves the superiority of this method.

Dr. Thos. P. Hinman described the process of stamping aluminum crowns with the Morrison outfit. After the crowns are fitted and polished he thickens up the cusps with amalgam. These serviceable crowns can be stamped up in a few moments, and for twenty cents you can buy enough aluminum plate to make a hundred crowns. Take the measure of the tooth with a tightly twisted wire, and an accurate fit is easily secured. The great advantage of aluminum crowns is in cheapness of material, ease, and rapidity of manufacture, and the fact that they never tarnish.

Dr. Rosser prefers making a crown with a band, into which he can look and assure himself of its accurate fit.

Dr. Johnson thinks the crowns stamped with the Morrison mandrels only suited to very symmetrical teeth with good straight walls, but that a band can be adjusted more accurately before the cap is added than after. The mandrels, though of different sizes, are all of one shape, while teeth are infinite in variety. In shaping a seamless crown with contouring pliers the shape of the cusps is liable to be injured, and in filling in the cusps with solder there is danger of the solder flowing up the sides of the crown and spoiling the fit. If the cusps are filled in solidly, and the band adjusted accurately before the two are joined, there is more certainty of success. In filling in a seamless crown with solder you can't make the surface of the solder in the crown conform to the surface of the end of the root.

Dr. Thompson puts wax in the crown and then places it on the root, which is forced into the wax, showing exactly where to grind the root to fit the surface of the solder.

Dr. Catching paints the surface with Spanish whiting and water, which prevents the solder from flowing where it is not wanted.

Dr. W. H. Morgan spoke of the proper preparation of borax for soldering. It should be heated to drive off the water of crystallization which causes the solder to "crawl." Borax absorbs moisture from the atmosphere, and when heated it forms steam and puffs up the borax, lifting off the solder. Dr. Morgan thinks that many crowns fail because they are set too short, a portion of the neck of the tooth being left uncovered. It is better to make the crown too long and separate the gum from the tooth than to make it too short. When teeth that have lost their natural support and become loose in the socket are properly crowned, covering all portions from which the peridental membrane has been removed, affording protection against the ingress of foreign substances, this source of constant irritation being removed, the teeth will assume healthy conditions and often become firm again without any other treatment than the crowning. "I never hesitate now," he said, "to put a crown in a root, however loose it may be in the socket."

Dr. Crenshaw had used the "J. J. R. Patrick outfit" a great deal, but has now discarded it in favor of the ready-made seamless crowns on the market. They are so easily adapted and demand so much less time. Get them too large if anything, and make the necessary change with a reducing die. Never attempt to stretch a crown that is too small. They are especially available as dummies for masticating wedges. The crowns should be of 23 or 24 karat gold, filled in with 22 karat solder, and set in position with 20 or even 18 karat solder.

Dr. Johnson had recently seen the failure of what was otherwise a beautiful piece of work, because the crown on a wisdom tooth had been made too short. The cement had dissolved out almost entirely. The centrals had porcelain crowns and the bridge ran back to the wisdom teeth on each side. The other crown had worn through from not having sufficient solder in the cusps.

Dr. Browne: In making a Richmond crown, when the porcelain is put in position, there is often a space between it and the band. If this space is packed with pellets of gold, solder will flow over it, and no place be left for deposits to lodge.

Dr. H. H. Johnson always backs a porcelain tooth with pure gold, bringing it down to a very thin edge.

For bicuspids Dr. Catching uses a Logan crown with a gold band.

Dr. Thompson uses a Logan crown with a plate ferrule, covering the latter with body, and baking in a Downie furnace. You can get all shades of body and want merely a film to hide the gold band. It makes a beautiful tooth, looking as if growing from under the gum.

Dr. D. D. Atkinson thought that sufficient stress had not been laid on the importance of having heavy cusps. The weak point in a gold crown is the liability to wear through. He does not see how enough solder can be flowed into the cusps of a seamless crown to enable it to resist the force of mastication. The cusps should be made thick enough to fill all the space between the occluding tooth and the stump.

Dr. Browne: If you use an asbestos film for investment, you can fill a seamless crown entirely full of solder if you want to. With this material for investment, you don't have to wait, as you do for plaster, to dry out. As the wax melts, the fiber becomes hard and holds the crown rigidly in position, and you can apply solder at once. It hugs closely and stays where you put it.

Dr. Morgan has been using asbestos for forty years. but always mixed with plaster and waited for it to dry out.

Dr. Browne: I use it *just so*, and do not have to wait any.—*Items of Interest.*

